# Turkish coffee has an antitumor effect on breast cancer cells in vitro and in vivo

**DOI:** 10.1186/s12986-024-00846-4

**Published:** 2024-09-13

**Authors:** Mohamed N. Amin, Usama Ramadan Abdelmohsen, Yara A. Samra

**Affiliations:** 1 Biochemistry Department, Faculty of Pharmacy, Mansoura University, Mansoura, 35516 Egypt; 2 Department of Pharmacognosy, Faculty of Pharmacy, Minia University, Minia, 61519 Egypt; 3 Department of Pharmacognosy, Faculty of Pharmacy, Deraya University, New Minia City, 61111 Egypt; 4 Department of Basic Medical Sciences, Faculty of Oral and Dental Medicine, Ahram Canadian University, Giza, 12566 Egypt

**Keywords:** Breast cancer, Coffee extract, Fridamycin-H, Caspase-8, PPAR-γ, Β-catenin

## Abstract

**Background:**

Breast cancer is the most diagnosed cancer in women. Its pathogenesis includes several pathways in cancer proliferation, apoptosis, and metastasis. Some clinical data have indicated the association between coffee consumption and decreased cancer risk. However, little data is available on the effect of coffee on breast cancer cells in vitro and in vivo.

**Methods:**

In our study, we assessed the effect of Turkish coffee and Fridamycin-H on different pathways in breast cancer, including apoptosis, proliferation, and oxidative stress. A human breast cancer cell line (MCF-7) was treated for 48 h with either coffee extract (5% or 10 v/v) or Fridamycin-H (10 ng/ml). Ehrlich solid tumors were induced in mice for in vivo modeling of breast cancer. Mice with Ehrlich solid tumors were treated orally with coffee extract in drinking water at a final concentration (v/v) of either 3%, 5%, or 10% daily for 21 days. Protein expression levels of Caspase-8 were determined in both in vitro and in vivo models using ELISA assay. Moreover, P-glycoprotein and peroxisome proliferator-activated receptor gamma (PPAR-γ) protein expression levels were analyzed in the in vitro model. β-catenin protein expression was analyzed in tumor sections using immunohistochemical analysis. In addition, malondialdehyde (MDA) serum levels were analyzed using colorimetry.

**Results:**

Both coffee extract and Fridamycin-H significantly increased Caspase-8, P-glycoprotein, and PPAR-γ protein levels in MCF-7 cells. Consistently, all doses of in vivo coffee treatment induced a significant increase in Caspase-8 and necrotic zones and a significant decrease in β- catenin, MDA, tumor volume, tumor weight, and viable tumor cell density.

**Conclusion:**

These findings suggest that coffee extract and Fridamycin-H warrant further exploration as potential therapies for breast cancer.

## Introduction

Coffee is one of the most popular beverages in the world, and a common constituent of our daily diet. Coffee is a heterogeneous mixture of phytochemicals belonging to different categories. Its main constituents are caffeine, polyphenols, lignans, melanoidins, alkaloids, terpenoids, minerals, vitamins, and insoluble fibers. Some coffee constituents have been identified as powerful antioxidants, such as caffeine and chlorogenic acids [1].

Many components of our daily diet significantly increase or decrease the risk of breast cancer, such as sweets, white bread, vegetables, and fruits. Hence, changing dietary styles has a great impact on the prevention of breast cancer and/or achieving a better quality of life for patients [2]. Many of these dietary components are of plant origin, which raises the importance of phytochemicals, bioactive secondary metabolites originating from plants [3]. The main treatment methods for breast cancer include radiotherapy, chemotherapy, and surgery [4]. However, current treatment methods have significant side effects and drug resistance. Many phytochemicals reportedly help prevent and treat breast cancer [5]. These phytochemicals might affect cancer cell apoptosis, proliferation, metastasis, and angiogenesis [6].

Many clinical studies have indicated the protective effect of coffee consumption in breast cancer [7–9] or the association of high postdiagnostic coffee consumption with better breast cancer and overall survival [10]. Similar results were obtained by meta-analysis studies [11].

However, some clinical studies indicated no association between breast cancer risk and increased coffee consumption [12, 13]. Also, little data is available about the effect of coffee consumption on breast cancer cells in vitro and in vivo [14]. Hence, more experimental data are needed to clarify the possible effects of coffee consumption on breast cancer cells.

Another source of bioactive secondary metabolites is marine sponge-derived actinomycetes, which are a source of many chemical compounds with diverse pharmacological activities such as antioxidant, cytotoxic, and antibacterial effects [15]. Moreover, marine sponges are considered a source of many secondary metabolites with antitumor activity [16]. Some of these compounds induce apoptosis in breast cancer-stem cells in vitro, leading to antitumor effect [17]. Moreover, some marine actinomycetes-derived secondary metabolites decreased tumor necrosis factor-related apoptosis-inducing ligand (TRAIL)-induced resistance to apoptosis in breast cancer cells [18].

Breast cancer is the most diagnosed cancer between women [19, 20]. Therefore, modern medicine’s target is to improve the early detection, treatment, and prevention of breast cancer [21]. Breast cancer pathogenesis includes several pathways that participate in cancer proliferation, apoptosis, and metastasis.

Insulin-like growth factor binding protein-3 (IGFBP-3) is a multifunctional protein that participates in the pathophysiology of several human diseases, including cancer. Several inhibitors for cell growth in human cancer cells increase IGFBP-3 expression. It is the main transporter of insulin growth factors (IGFs) in circulation. Also, IGFBP-3 is a p53 tumor suppressor-regulated protein [22, 23]. Several studies have shown that IGFBP-3 has both tumor-suppressing and tumor-promoting effects according to the posttranslational modifications, assay methods, and cell types [24].

P-glycoprotein is a membrane transporter that can efflux drug molecules outside the cancer cell, decreasing chemotherapeutic efficacy. Cancer cells upregulate P-glycoprotein expression as an adaptive response to avoid cell death during chemotherapy [25].

Peroxisome proliferator-activator receptor-γ (PPARγ) is a nuclear receptor that is expressed in breast, prostate, and colon cancer cells. Its ligands have been evidenced to inhibit cellular proliferation [26, 27]. Therefore, PPARγ acts as a tumor suppressor. Cancer cells usually show decreased expression of PPARγ and increased expression of canonical Wnt/β-catenin.

Understanding the exact effect of coffee on breast cancer cells and the underlying mechanisms would be beneficial for adjustment of dietary recommendations in breast cancer patients and post treatment survivors. Therefore, we aimed to investigate the effect of coffee on breast cancer cells in vitro and in vivo.

We have isolated a new bioactive molecule from the strain Actinokineospora spheciospongiae, namely Fridamycin-H [15]. The new isolated compound showed promising antitrypanosomal activity, but its effect on cancer cells has not yet been explored. Hence, we assessed the effect of Fridamycin-H on apoptotic and proliferation markers in the MCF-7 cell line.

In the present study, we aimed to investigate the effect of coffee extract and Fridamycin-H on MCF-7 cell line through the assessment of an apoptotic marker, caspase-8, cell proliferation markers PPARγ and IGFBP-3, and a drug resistance marker, P-glycoprotein. Also, we investigated the effect of the coffee extract on an Ehrlich solid tumor model through the assessment of apoptotic marker caspase-8, cell proliferation markers β-catenin and PPARγ, and an oxidative stress marker, malondialdehyde (MDA).

## Materials & methods

### Coffee extract preparation

Turkish coffee (Abu Auf, Cairo, Egypt; *Coffea arabica*) was prepared using the usual Turkish coffee preparation procedure. Briefly, 12 gm coffee was mixed with 500 ml distilled water, heated till near boiling (~ 90 °C), and filtered with Whatmann No.1 filter paper. Then, the filtrate was sterilized by bacterial filtration. The sterile filtrate, called coffee extract, was used for in vitro and in vivo experiments at different (v/v) concentrations.

### Fridamycin-H

Fridamycin-H was previously isolated from *Actinokineospora spheciospongiae* sp. nov. (DSM 45935T, GeneBank accession no. GU318361) cultivated from the Red Sea sponge *Spheciospongia vagabunda.* The sponge was collected from offshore Ras Mohamed, Egypt (GPS: 27°47.655 N; 34°12.904 W) by SCUBA diving in August 2006 [15].

### Cell culture

A human breast cancer cell line (MCF-7) was obtained from ATCC via Nawah Scientific, Cairo, Egypt. The cells were maintained in Dulbecco’s modified eagle’s medium (DMEM; LONZA, UK) in a 5% CO_2_ and 37 °C incubator. The medium was supplemented with 100 U/ml penicillin, 100 µg/ml streptomycin, and 10% fetal bovine serum (FBS, GIBCO, UK). Medium was changed every other day, and cells were trypsinized and sub-cultured at 90% confluency. Cell seeding was performed in a 6-well plate (10^5^ cells/well) and maintained till 80% confluent. Then, the medium was changed to DMEM with 2% FBS, and experiments were conducted. Some cells were untreated to be used as a control (MCF-7); other cells were treated with coffee extract at a final concentration (v/v) of 5% (MCF-7 + 5%) or 10% (MCF-7 + 10%). Also, some cells were treated with an aqueous fraction of Fridamycin-H at a final concentration of 10 ng/ml (MCF-7 + Frida.-H). After 48 h of incubation, cell lysate and supernatant were collected in each group for protein expression analysis.

### Animals

Female Swiss albino mice weighing 20–25 g were purchased from the Egyptian Organization for Biological Products and Vaccines. All animals were kept in a 12-hour light-dark cycle at room temperature, with water ad libitum and a standard diet. The study was performed according to the ethical guidelines for using laboratory animals, and the Research Ethics Committee-Faculty of Pharmacy-Mansoura University approved the study protocol (2023 − 218).

### Ehrlich solid tumor model

Ehrlich ascites cells were injected in female Swiss albino mice at 7–10 days intervals using serial i.p. passage [28]. Seven days after injection, mice were euthanized, and the peritoneal cavity was used for aspiration of ascites fluid. Normal saline was used to wash the aspirated cells three times; then, they were centrifuged. The Trypan blue method was used to determine viable cell count. Finally, cells were suspended in normal saline at a final cell concentration of 5 × 10^5^/100 µl.

Mice were injected with 100 µl cell suspension/mouse subcutaneously in the right hind limb (thigh). Some mice were either kept untreated to serve as a control group (Control; *n* = 5) or treated orally with 10% (v/v) coffee extract in daily water for 21 days (Control + 10%; *n* = 5). Day “0” was set at 5 days after tumor implantation. At day “0” the primary tumor size was 50–100 mm^3^. On day 1, animals were distributed into six groups (*n* = 7). Some mice bearing Ehrlich solid tumor were untreated (EAC), while others were treated orally with coffee extract in the drinking water at a final concentration (v/v) of either 3% (EAC + 3%), 5% (EAC + 5%) or 10% (EAC + 10%) daily for 21 days. The tumor volume was measured at days 0, 5, 10, 15, 20 (*n* = 7). Solid tumor volume was measured via digital caliper using the equation solid tumor volume = largest diameter x its perpendicular^2^ × 0.5) [29]. Mice were sacrificed in each group after 21 days for protein marker analysis. The percentage relative change in tumor volume ΔT/ΔC (%) and % inhibition in tumor size were calculated where ΔT/ΔC (%)=ΔT/ΔC × 100% (ΔT = tumor volume change of the coffee extract-treated group, ΔC = tumor volume change of the EAC group on the final day of the study) and % Inhibition = 100-ΔT/ΔC. Then, blood was collected, serum was separated, and the tumor mass was isolated and weighed. Tumors were divided into two parts; one was lysed and used for protein expression level assay using ELISA, and the other part was preserved in buffered formalin and processed for histopathological and immunohistochemical investigations.

### Hematoxylin & eosin staining

Tumors were isolated, fixed in formalin (10% buffered), and implanted in paraffin. Hematoxylin & Eosin (H&E) staining for 5-µm sections was performed. Immunohistochemical and histological examination was performed for three mice in each group. Histopathological and morphological changes were blindly examined with light microscopy (Olympus BH-2, Tokyo, Japan) combined with a digital camera (Nikon, Japan).

### Immunohistochemical analysis

β-catenin expression was detected by immunohistochemical analysis in 5-µm solid tumor sections. Paraffin was removed from the slides by xylene. Slides were incubated overnight with anti-mouse IgG monoclonal antibody (Dako, IR702) at 4º C. Slides were washed with 1x phosphate buffer saline (PBS). The detection was performed using Poly Detector Plus DAB HRP Brown Detection System (Bio SB, BSB 0259). Sections were viewed using a light microscope with a digital camera-aided computer system.

### ELISA, caspase-8, & malondialdehyde assay

Collected cells from in vitro experiments were washed three times with precooled 1x PBS. Then the cells were lysed using three freeze-thaw cycles at − 20 °C. The cell lysate was centrifuged for 10 min at 1,500 x g at 4 °C to remove cell debris. Commercially available ELISA kits were used to analyze cell lysate protein expression levels of P-glycoprotein (MyBioSource, CA, #MBS729657), PPAR-γ (MyBioSource, CA, #MBS2503174), and Caspase-8 (MyBioSource, CA, #MBS267173) according to the manufacturer’s instructions. Collected cell culture media was centrifuged at 1,000 x g for 15 min, then IGFBP-3 was determined using a commercially available ELISA kit (MyBioSource, CA, #MBS732160) according to the manufacturer’s instructions.

As for tissue lysate preparation, mice tissue samples were chopped, washed with PBS, homogenized in PBS (tissue weight (g): PBS (mL) volume = 1:9), and centrifuged at 5,000 x g for 5 min. Tissue lysates were used for the ELISA assay of mouse Caspase-8 (Novus Biologicals, CO, #NBP2-75040) and IGFBP-3 (MyBioSource, CA, #MBS2502084) using commercially available kits according to the manufacturer’s instructions. According to the manufacturer’s instructions, MDA was analyzed in serum using a commercially available kit (Biodiagnostic, Egypt, #MD2529).

### Statistical analysis

Statistical analysis was performed using IBM SPSS version 25 (Chicago, IL, USA) and Microsoft Excel (Microsoft^®^OfficeExcel^®^2016). Data were presented as means ± SEM. Data were analyzed using a one-way ANOVA test followed by Tukey’s posthoc test for inter-group comparison. Statistical significance was *P* < 0.05.

## Results

### Coffee extract induced a dose-dependent elevation in Caspase-8, P-glycoprotein & IGFBP-3 in MCF-7

**Fig. 1:**
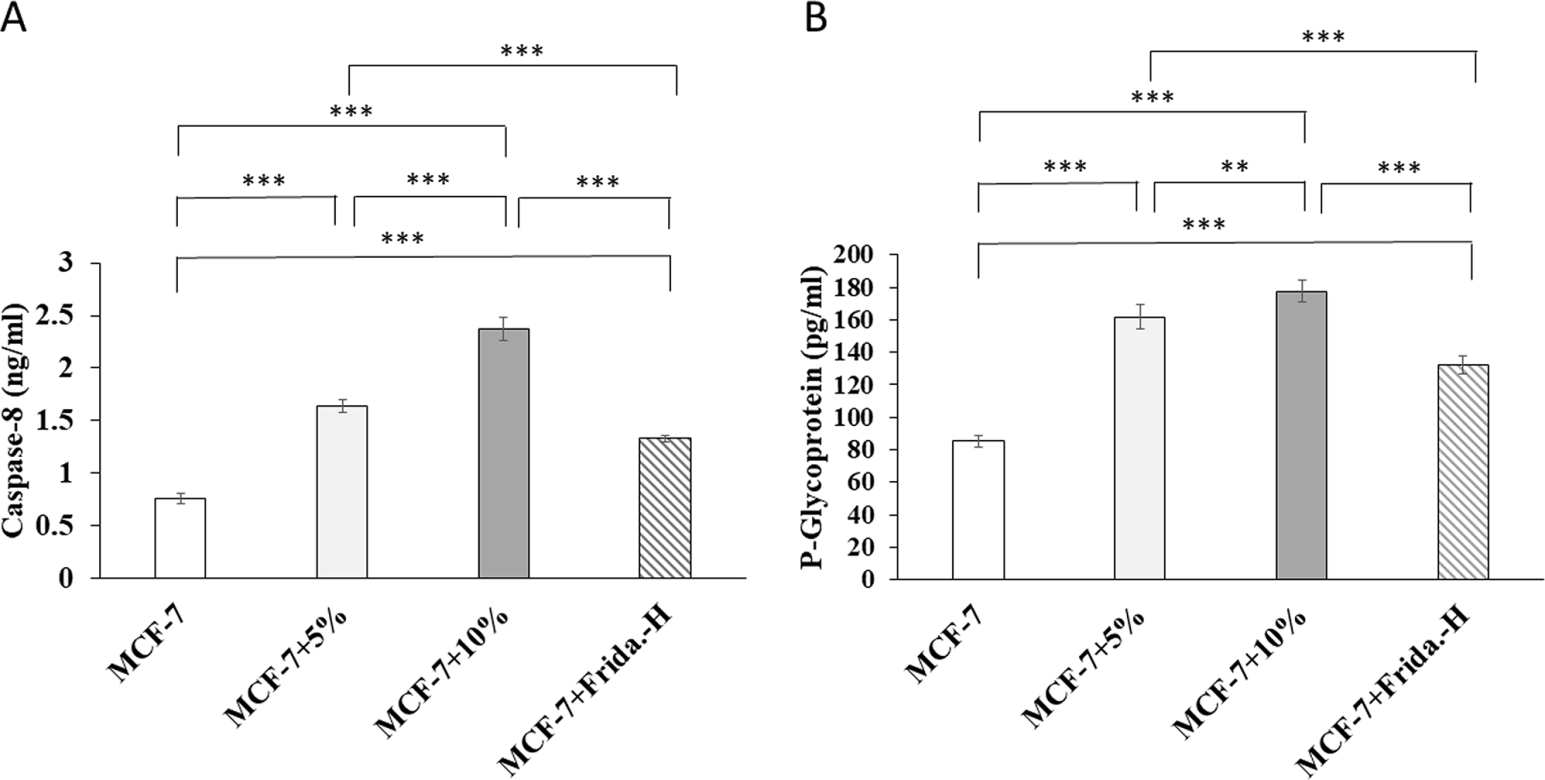
Protein expression levels of Caspase-8 (**A**) and P-glycoprotein (**B**) in cell lysate of untreated breast cancer cell line (MCF-7), MCF-7 treated for 48 h with 5% (V/V) coffee extract (MCF-7 + 5%), MCF-7 treated for 48 h with 10% (V/V) coffee extract (MCF-7 + 10%), and MCF-7 treated for 48 h with Fridamycin-H (MCF-7 + Frida.-H). (_**_): very significant difference (P˂0.01); (_***_): highly significant difference (P˂0.001)

**Fig. 2:**
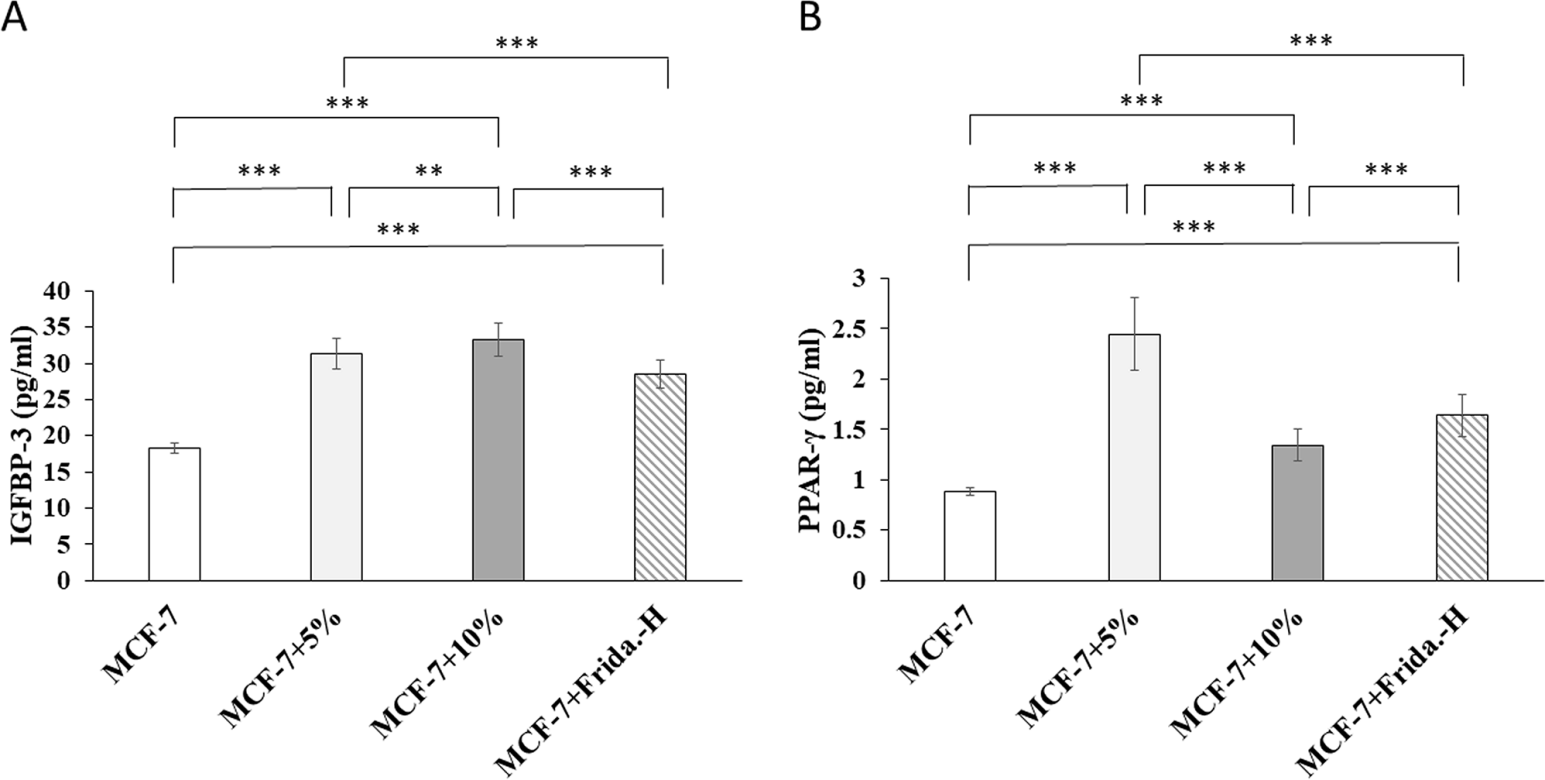
Protein expression levels of insulin-like growth factor binding protein-3 (IGFBP-3) in cell supernatant (**A**) and peroxisome proliferator-activated receptor gamma (PPAR-γ) in cell lysate (**B**) of untreated breast cancer cell line (MCF-7), MCF-7 treated for 48 h with 5% (V/V) coffee extract (MCF-7 + 5%), MCF-7 treated for 48 h with 10% (V/V) coffee extract (MCF-7 + 10%), and MCF-7 treated for 48 h with Fridamycin-H (MCF-7 + Frida.-H). (_**_): very significant difference (P˂0.01); (_***_): highly significant difference (P˂0.001)

Caspase-8 (Fig. 1A), P-glycoprotein (Fig. 1B), and IGFBP-3 (Fig. 2A) protein levels significantly increased in MCF-7 + 5%, MCF-7 + 10%, and MCF-7 + Frida.-H groups as compared to MCF-7 (P˂0.001). The coffee extract increased Caspase-8, P-glycoprotein, and IGFBP-3 protein levels in a dose-dependent manner. MCF-7 + 10% group showed a significantly higher Caspase-8 (P˂0.001), P-glycoprotein (P˂0.01), and IGFBP-3 (P˂0.01) protein expression as compared to MCF-7 + 5% group with a 0.44 fold increase in Caspase-8. Protein expression levels of PPAR-γ (Fig. 2B) significantly increased in MCF-7 + 5%, MCF-7 + 10%, and MCF-7 + Frida.-H groups compared to MCF-7 (P˂0.001). However, MCF-7 + 10% cells showed significantly lower PPAR-γ levels than MCF-7 + 5% (P˂0.001).

### The coffee extract increased caspase-8 and decreased IGFBP-3 in a dose-dependent manner in ehrlich solid tumors

**Fig. 3:**
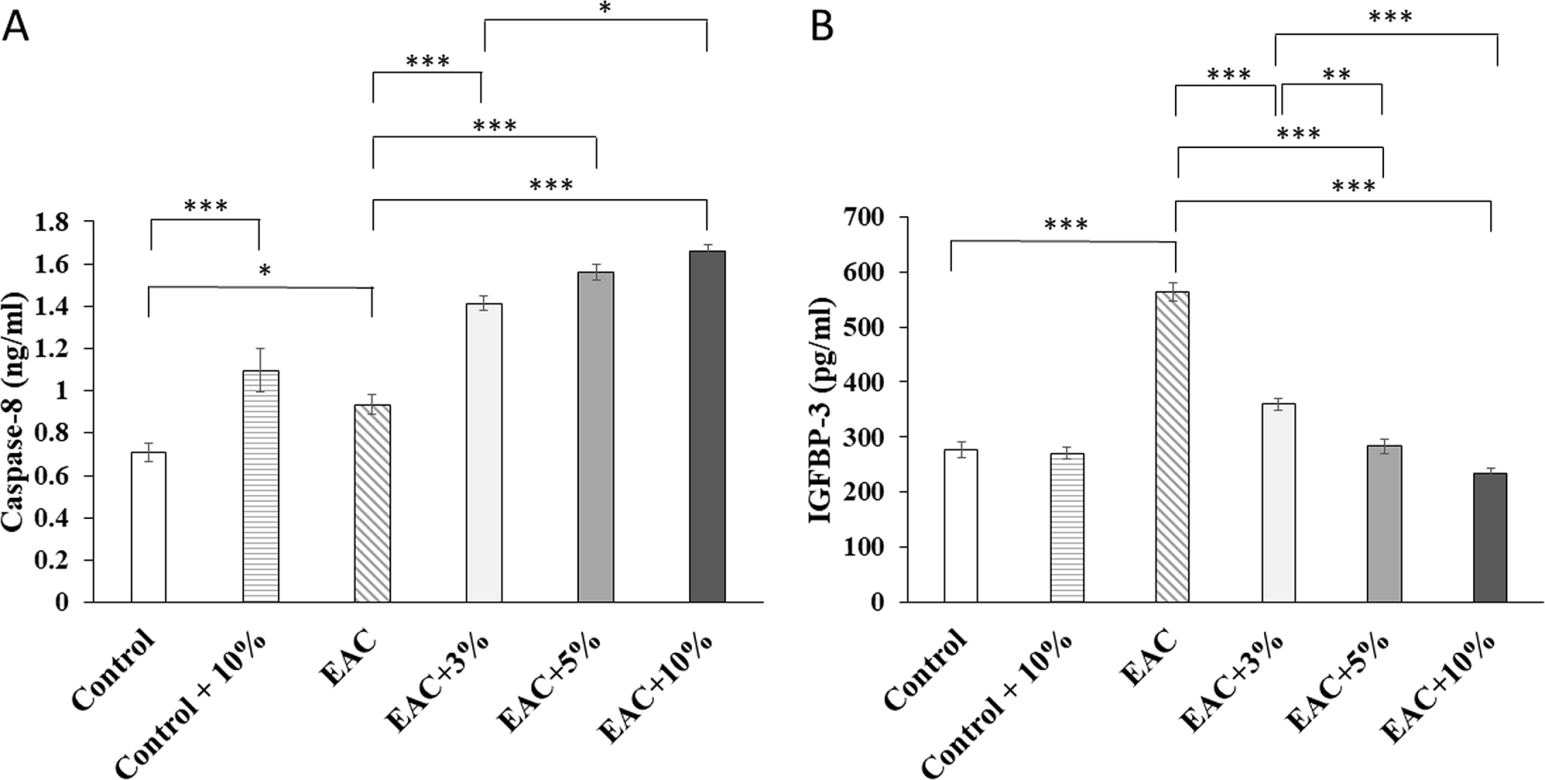
Protein expression levels of Caspase-8 (**A**) and insulin-like growth factor binding protein-3 (IGFBP-3) (**B**) in right hind limb skeletal muscle tissue lysate of wild type mice (Control), wild type mice treated daily for 21 days with 10% (V/V) coffee extract (Control + 10%), untreated mice with solid Ehrlich ascites carcinoma (EAC), and EAC mice treated daily for 21 days with either 3% (V/V) coffee extract (EAC + 3%), 5% (V/V) coffee extract (EAC + 5%), or 10% (V/V) coffee extract (EAC + 10%). (*): significant difference (P˂0.05); (**): very significant difference (P˂0.01); (***): highly significant difference (P˂0.001)

Caspase-8 (Fig. 3A) levels were significantly higher in EAC + 3%, EAC + 5%, and EAC + 10% groups than EAC group (P˂0.001). Similar to in vitro results, Caspase levels increased in EAC + 3%, EAC + 5%, and EAC + 10% groups in a dose-dependent pattern. The EAC + 10% group showed a significant increase (P˂0.05) in Caspase-8 as compared to EAC + 3%. EAC + 3%, EAC + 5%, and EAC + 10% showed 0.51, 0.67, and 0.77-fold increase in Caspase-8 as compared to EAC group; respectively. Also, coffee extract increased Caspase-8 levels significantly (P˂0.001) in the Control + 10% compared to the Control group.

IGFBP-3 (Fig. 3B) levels were significantly lower in EAC + 3%, EAC + 5%, and EAC + 10% groups than EAC group (P˂0.001). In contrast to in vitro results, IGFBP-3 levels decreased in EAC + 3%, EAC + 5%, and EAC + 10% groups in a dose-dependent pattern. EAC + 3% group showed a significant increase in IGFBP-3 as compared to EAC + 5% (P˂0.01) and EAC + 10% (P˂0.001). EAC + 3%, EAC + 5%, and EAC + 10% showed 0.36, 0.49, 0.58-fold decrease in IGFBP-3 as compared to EAC group; respectively.

**Fig. 4:**
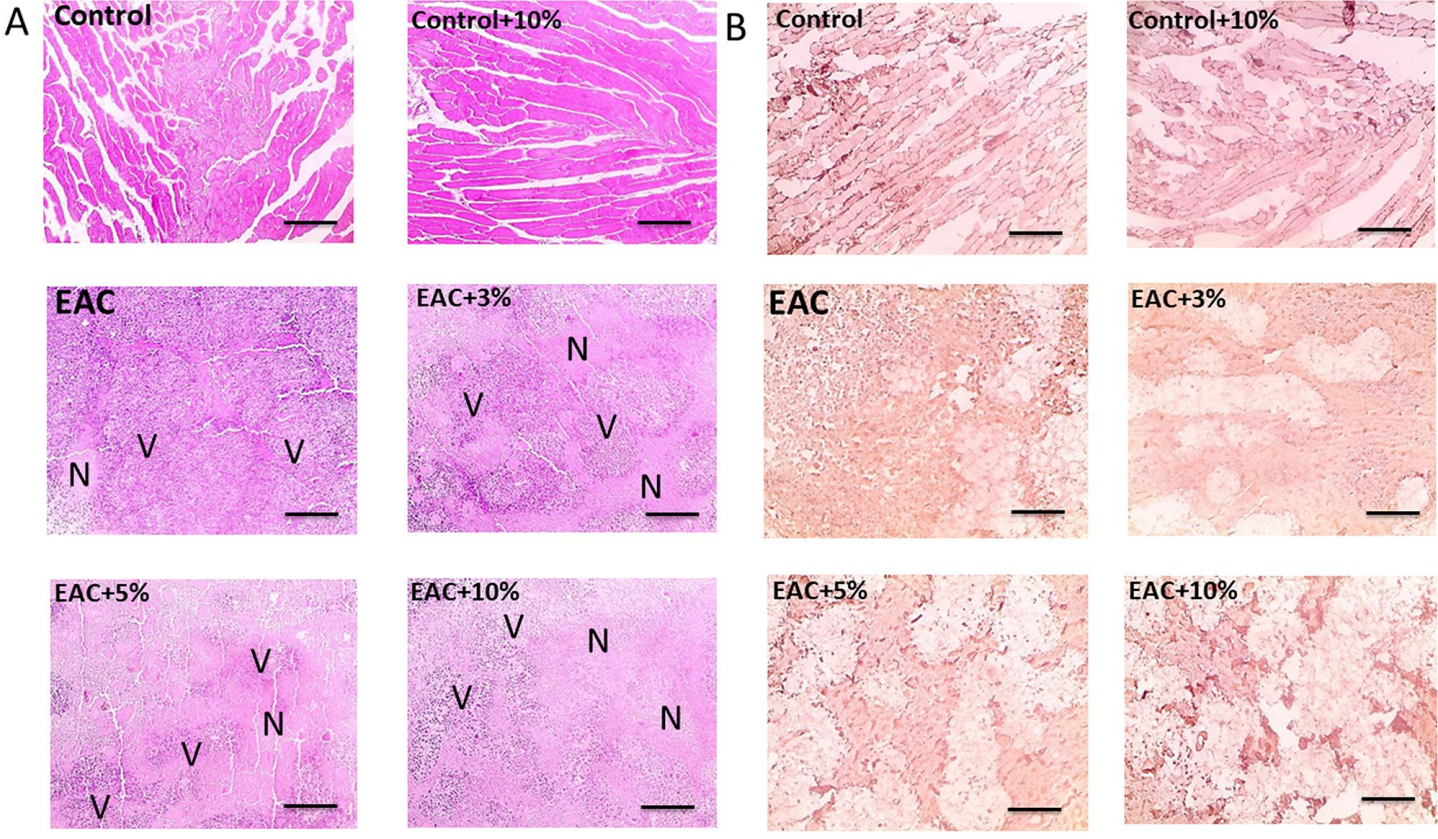
Microscopic images of hematoxylin and eosin stained sections (**A**) showing normal skeletal muscles in the untreated mice control group (Control) and wild mice treated with 10% (V/V) coffee extract (Control + 10%). Sections from mice with solid Ehrlich ascites carcinoma (EAC) tumor masses show wide viable areas (V) and mitotic figures interspaced by small necrotic zones (N). In comparison, EAC mice treated daily for 21 days with coffee extract at a final concentration (V/V) of either 3% (EAC + 3%), 5% (EAC + 5%), or 10% (EAC + 10%) showed an increase in necrotic zones with a decrease in the density of viable tumor cells in a dose-dependent manner. Microscopic images of immunohistochemistry-stained sections against β- catenin (**B**) showing negative staining in the Control and Control + 10% groups. EAC group showed a positive β- catenin signal. β- catenin protein expression level gradually decreased by increasing the coffee extract daily dose, as seen in the EAC + 3%, EAC + 5%, and EAC + 10% groups. Scale Bar: 100 μm

### Coffee extract increased necrotic zones and decreased β-catenin expression in Ehrlich solid tumors

Microscopic images of H&E-stained sections (Fig. 4A) showed normal skeletal muscles in Control and Control + 10% groups. Sections from the EAC group showed wide viable areas consisting of numerous large, round, and polygonal deeply stained tumor cells with pleomorphic shapes, hyperchromatic nuclei, and mitotic figures interspaced by small necrotic zones and mitotic figures. The neoplastic cells invaded and destroyed adjacent skeletal muscles. In comparison, EAC + 3%, EAC + 5%, and EAC + 10% showed an increase in necrotic zones with a decrease in the density of viable tumor cells in a dose-dependent manner.

Converesely, immunohistochemistry-stained sections against β- catenin (Fig. 4B) showed negative staining in the Control and Control + 10% groups. EAC group showed a positive β- catenin signal as evidenced by a strong brown staining. β- catenin protein expression level gradually decreased by increasing the coffee extract daily dose, as seen in the EAC + 3%, EAC + 5%, and EAC + 10% groups.

### Coffee extract significantly decreased Ehrlich solid tumor volume and weight

**Fig. 5:**
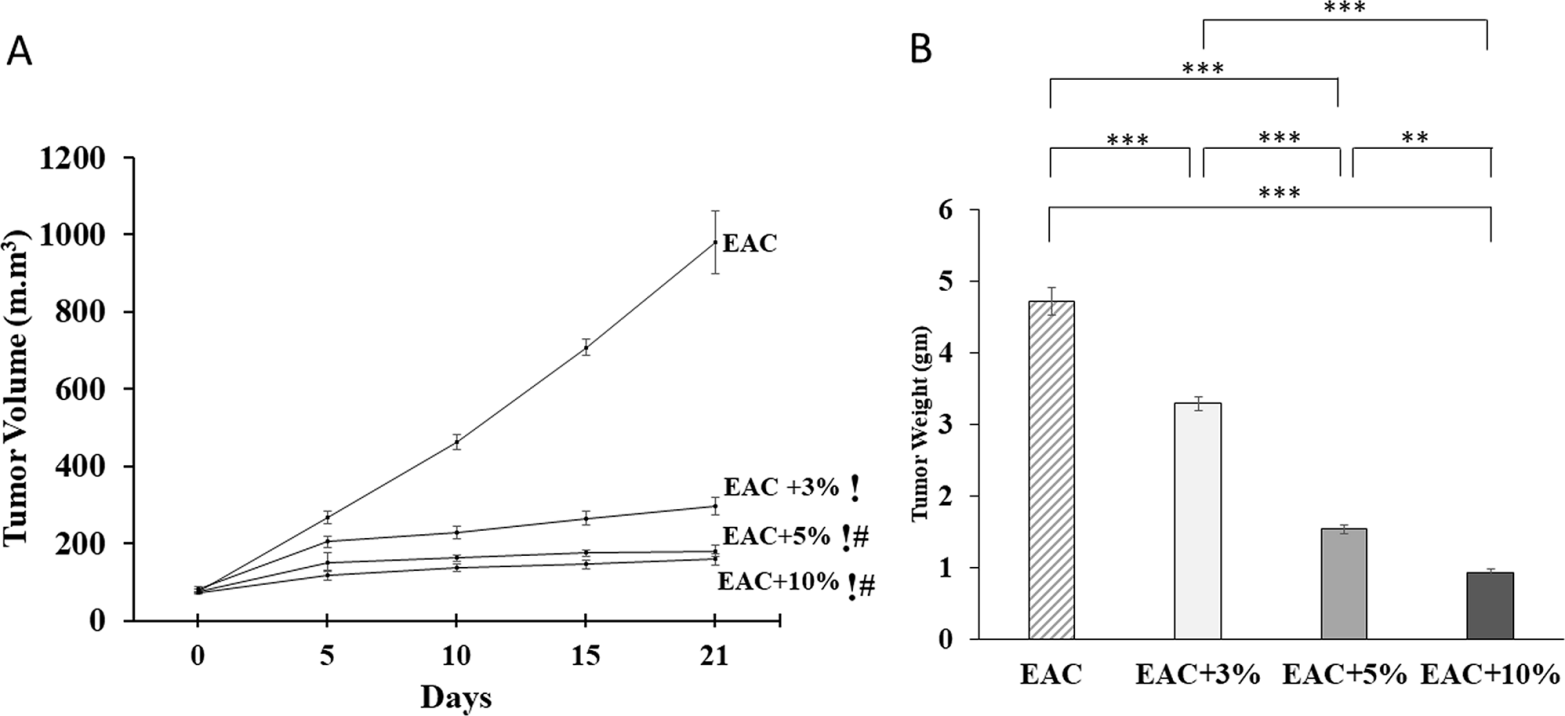
Tumor volume (**A**) in solid Ehrlich tumor-bearing mice. Ehrlich ascites carcinoma cells were injected into mice. Day 5 after injection was considered Day 0. The solid tumors’ volume was measured every fifth day till the end of the experiment in untreated mice bearing solid Ehrlich tumor (EAC), and EAC treated daily for 21 days with coffee extract at a final concentration (V/V) of either 3% (EAC + 3%), 5% (EAC + 5%), or 10% (EAC + 10%). (!): highly significant difference as compared to the EAC group (*P* < 0.001). (#): highly significant difference as compared to EAC + 3% group (*P* < 0.001). Tumor weight (**B**) at the end of the experiment in EAC, EAC + 3%, EAC + 5%, and EAC + 10%. (**): very significant difference (P˂0.01). (***): highly significant difference (P˂0.001)

Tumor volumes (Fig. 5A) on day 21 were significantly lower in the EAC + 3%, EAC + 5%, and EAC + 10% groups than the EAC group (P˂0.001). Moreover, tumor volume on day 21 in EAC + 5%, and EAC + 10% were significantly lower than that of the EAC + 3 group (P˂0.001). The percentage inhibition (Table 1) in tumor size was 76.08, 88.46, 90.44 in EAC + 3%, EAC + 5%, and EAC + 10%, respectively. Consistently, tumor weight (Fig. 5B) on day 21 in EAC + 3%, EAC + 5%, and EAC + 10% groups decreased significantly compared to EAC group (P˂0.001). The decrease in tumor weight was gradual with increasing coffee extract doses. The EAC + 3% group showed a significant increase in tumor weight (P˂0.001) compared to the EAC + 5% and EAC + 10% groups. Also, the EAC + 5% group showed a significant increase in tumor weight (P˂0.001) compared to EAC + 10%.

**Table 1:** Tumor volume changes and % inhibition in tumor size induced by coffee extract

	Tumor volume (m.m^3^)		ΔT/ΔC (%)	% inhibition
	Day 0	Day 21		
EAC	75.65 ± 5.49	980.54 ± 30.88	100	0
**EAC + 3%**	**80.77** **± 5.63**	**297.21** **± 8.42**	**23.91**	**76.08**
**EAC + 5%**	**76.37** **± 6.21**	**180.74** **± 6.08**	**11.53**	**88.46**
**EAC + 10%**	**71.97** **± 4.05**	**158.42** **± 5.17**	**9.55**	**90.44**

EAC: Ehrlich solid tumor group; EAC + 3%, EAC + 5%, and EAC + 10%: EAC group treated with 3, 5, or 10% (v/v) coffee extract for 21 days; Day 0 = 5 days after mice i.P. injection of Ehrlich carcinoma cells; ΔT/ΔC (%) = ΔT/ΔC × 100% (ΔT = tumor volume change of the coffee extract-treated group, ΔC = tumor volume change of the EAC group on the final day of the study); % inhibition = 100 − ΔT/ΔC

**Fig. 6:**
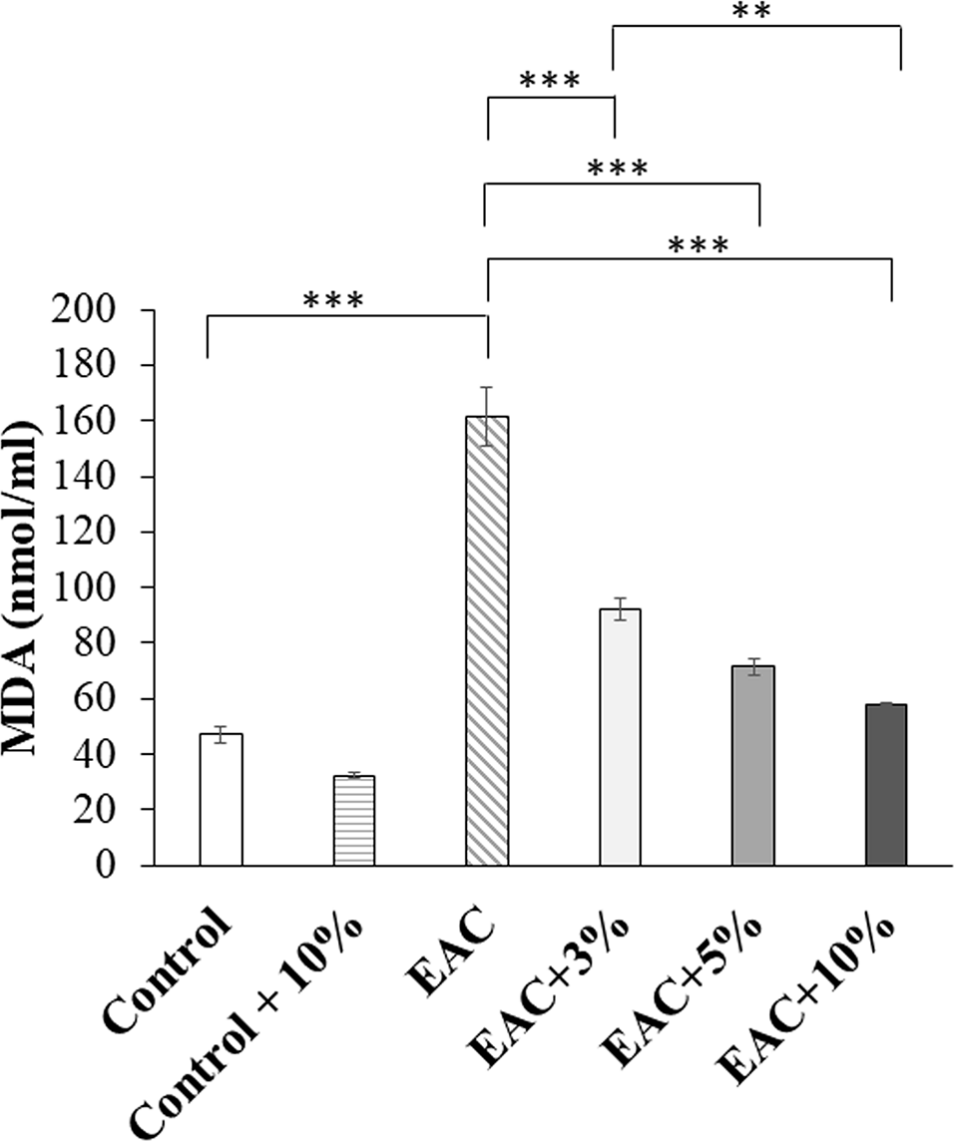
Malondialdehyde serum levels at the end of experiment in untreated mice (Control) group, control group treated daily for 21 days with 10% (V/V) coffee extract (Control + 10%), untreated mice bearing solid Ehrlich tumor (EAC), and EAC treated daily for 21 days with coffee extract at a final concentration (V/V) of either 3% (EAC + 3%), 5% (EAC + 5%), or 10% (EAC + 10%). (**): very significant difference (P ˂ 0.01). (***): highly significant difference (P ˂ 0.001)

### Coffee extract significantly decreased oxidative stress in Ehrlich solid tumor in a dose-dependent manner

EAC + 3%, EAC + 5%, and EAC + 10% groups showed a significant decrease (P˂0.001) in serum MDA (Fig. 6) levels compared to the EAC group. Coffee extract’s effect on oxidative stress was dose-dependent. MDA levels in the EAC + 10% group were significantly lower than in EAC + 3% (P˂0.01). The EAC group showed a significant increase in MDA (P˂0.001) as compared to the Control group. However, the coffee extract had no significant effect on MDA levels in the Control + 10% compared to the Control group.

## Discussion

Previous studies have demonstrated that proliferation and apoptosis are two main pathways that participate in the pathogenesis of breast cancer [30, 31]. Several clinical studies have confirmed the association between coffee consumption and decreased cancer risk [32–36]. However, there is no clear data on the mechanisms by which coffee consumption might affect cancer cells. Hence, we aimed to investigate the effects of coffee extract on apoptosis (Caspase-8 and IGFBP-3), proliferation (PPAR-γ), and drug resistance (P-glycoprotein) markers in vitro using the MCF-7 cell line. In addition, we evaluated the effect of Fridamycin-H, a bioactive molecule that we previously isolated from *Actinokineospora spheciospongiae* [15], on these markers in MCF-7 cells. Also, we investigated the effect of coffee extract on apoptosis (Caspase-8 and IGFBP-3), proliferation (β- catenin), and oxidative stress (MDA) markers in vivo using an Ehrlich solid tumor model.

Impaired apoptosis is one of the hallmarks of cancer. Apoptosis is mainly regulated by Caspase-8 and Caspase-3. Caspase-8 initiates the apoptotic cascade. The dysfunction or reduced activity of Caspase-8 stimulates cellular proliferation, malignant transformation, and tumor progression. Also, Caspase-3 is activated via Caspase-8 signaling, which initiates cell death [37].

In our study, IGFBP-3 and Caspase-8 protein levels were significantly increased in MCF-7 treated with either coffee extract or Fridamycin-H compared to untreated MCF-7 cells. The coffee extract increased IGFBP-3 and Caspase-8 protein levels in a dose-dependent manner. These results suggest that coffee extract and Fridamycin-H might have a beneficial effect on breast cancer cells by affecting cell apoptosis.

Also, coffee extract significantly increased Caspase-8 dose-dependently in an Ehrlich solid tumor animal model. These results indicate that coffee extract can affect apoptosis in an in vivo model of breast cancer.

On the other hand, coffee extract decreased IGFBP-3 in the Ehrlich solid tumor animal model compared to untreated animals. IGFBP-3 might have tumor-suppressing or tumor-promoting effects depending on cell types, posttranslational modifications, and assay methods.

IGFBP-3 signaling participates in the pathogenesis of cancer. Numerous research has confirmed the antitumor effect of IGFBP-3 [24]. IGFBP-3 extends the half-life of IGFs by acting as a carrier in circulation for IGF-I and IGF-II [38, 39].

Several studies proved that the expression of IGFBP-3 is increased by various cell growth inhibitors, such as tumor suppressor gene p53 in human cancer cells [22, 23]. These studies proved that induction of IGFBP-3 by p53 activates apoptosis [40].

Additionally, many studies verified that IGFBP-3 inhibition by DNA methylation is associated with cancer development and resistance to chemotherapy and radiotherapy [41–43].

Previous studies have shown that the antiproliferative effect of IGFBP-3 in human breast cancer cells occurs via binding to cell surface proteins [44–46]. Also, IGFBP-3 activates Caspase-8 cleavage and induce apoptosis in breast cancer [47]. These results suggested that IGFBP-3 induces apoptosis and inhibits proliferation in tumors.

IGF’s effect might be activated or inhibited by IGFBP-3 according to IGFBP-3 concentration, type of the cell, cellular environment, and posttranslational modification [48–50]. The inhibitory effect of IGFBP-3 results from its competitive binding to IGFs. Consequently, it inhibits IGF’s activation of IGF receptors [39]. On the other hand, IGFBP-3 can increase IGF activity and concentration via binding to proteoglycans and heparin, thus working as a reservoir of IGFs [51, 52].

Cancer cells show resistance to different anticancer drugs, known as multidrug resistance (MDR) [53, 54]. MDR is considered the major challenge hindering the efficacy of chemotherapeutic agents. MDR effect is most challenging in metastatic tumors. MDR can be acquired during chemotherapeutic treatment of cancers [55, 56].

Adenosine triphosphate (ATP)-binding cassette (ABC) transporters are the main transporters involved in cancer MDR [57, 58]. ABC transporters proven to be associated with MDR include; MDR-associated protein 1 (MRP1), P-glycoprotein, and breast cancer resistance protein (BCRP) [59–61]. Such transporters lead to decreased cell drug accumulation and reduced drug efficacy.

The most well-known and first observed MDR-related transporter is P-glycoprotein. Hence, it is considered a primary target for MDR-overcoming therapy. Overcoming drug pumping outside the cancer cell is an important anticancer treatment approach. P-glycoprotein expression is increased in cancer cells, leading to drug efflux and evading chemotherapy-mediated cell death. [62–64].

High histological stage and grade carcinomas usually correlate with elevated expression of P-glycoprotein [65, 66]. The expression levels of these transporters usually increase after chemotherapy treatment.

In our study, P-glycoprotein expression increased in MCF-7 after treatment with coffee extract in a dose-dependent manner. Such a result might indicate that MCF-7 cells increased P-glycoprotein expression as an adaptive response to evade coffee extract-mediated cell death.

The PPAR family receptors are ligand-activated receptors in the nucleus, including PPARα, PPARγ, and PPARδ. PPAR present in adipose tissue is mainly PPARγ involved in lipid metabolism and adipocyte differentiation [67].

PPARγ ligands have been verified to inhibit cell proliferation in several types of cancer [68–70], including breast cancer, suggesting PPARγ’s role as a tumor suppressor. This was confirmed in rat models of mammary tumorigenesis, which showed that PPARγ agonists prohibited the progress of tumors [71]. The main targets of PPARγ are cell cycle regulators, such as cyclin D1 [72–74].

Canonical Wnt/β-catenin is upregulated, while PPARγ is downregulated in cancers, type 2 diabetes, and neurodegenerative diseases. PPARγ ligands inhibit the β-catenin pathway [73, 75], and the inhibited Wnt/β-catenin pathway activates PPARγ [76, 77]. Reportedly, nonsteroidal anti-inflammatory drug inhibition of β-catenin in tumor cells needs elevated expression of PPARγ [78].

β-catenin is an important intermediate in the Wnt pathway, and it interacts with E-cadherin that regulates cell-cell adhesion [79]. Nuclear accumulation of β-catenin causes breast cancer [80].

Nuclear β-catenin activates cyclin D1, which stimulates mammary hyperplasia. Also, nuclear β-catenin induces the expression of pro-invasive proteins [81]. Therefore, the subcellular distribution of β-catenin highly affects the phenotype and behavior of tumor cells. Increased cytoplasmic β-catenin might also be a marker for breast cancer due to the relationship between nuclear and cytoplasmic β-catenin in adenocarcinomas [82].

In our study, we report that coffee extract treatment increased necrotic zones and decreased β-catenin expression in an Ehrlich solid tumor animal model in a dose-dependent manner. Such results indicate that coffee extract might affect the proliferation of breast cancer cells via decreasing β-catenin expression.

Our results are consistent with many clinical studies indicating the protective effect of coffee consumption in breast cancer [7–9] or the association of high postdiagnostic coffee consumption with better breast cancer and overall survival [10]. Similar results were obtained by meta-analysis studies [11]. However, some clinical studies indicated no association between breast cancer risk and increased coffee consumption [12, 13]. However, these studies include all types of coffee consumption. Our data are based only on Turkish coffee.

Little literature indicates coffee extract’s in vitro effect against breast cancer. The antitumor effect of coffee extract against the MCF-7 cell line has been reported by Nigra et al.; however, they investigated the effect of Robusta coffee, not Arabica coffee [14]. The apoptotic effect of chlorogenic acid, a major constituent of coffee extract, against human breast cancer cell lines other than MCF-7 has been previously reported [83]. Commercially prepared coffee brews have shown apoptotic activity on human ovarian cancer cell lines [84].

Also, breast cancer risk increases via exposure to carcinogenic chemical compounds. Carcinogenic compounds lead to free radicals’ accumulation, which further damage biomolecules such as lipids. Lipid peroxidation increases MDA levels and gene mutations, which leads to breast cancer [85]. The free radical scavenging effect of coffee extract and its content of phenolics, especially chlorogenic acid, has been reported previously [86]. In our study, we measured the effect of coffee extract on MDA levels, and we found that coffee extract significantly decreased serum MDA levels in an Ehrlich solid tumor animal model in a dose-dependent manner.

## Conclusion

The present study suggests that coffee extract might affect apoptosis in breast cancer cells by increasing Caspase-8 levels, proliferation by reducing β-catenin expression, and oxidative stress by decreasing malondialdehyde (MDA) levels. These findings align with some clinical studies suggesting a protective effect of coffee consumption against breast cancer. Also, Fridamycin-H decreased Caspase-8 in breast cancer cells in vitro, suggesting it is a promising candidate for future in vivo studies in breast cancer models. Identifying the specific components within coffee extract responsible for the observed effects will be an important future plan. Isolating these bioactive compounds could lead to the development of targeted therapies.

## Data Availability

No datasets were generated or analysed during the current study.
